# Joint association of the newly proposed dietary index for gut microbiota and sleep disorders with survival among US adult population with diabetes and pre-diabetes

**DOI:** 10.1186/s12937-025-01162-0

**Published:** 2025-06-18

**Authors:** Ke Si, Chuanqin Shi, Yajing Huang, Chuanfeng Liu, Jingwei Chi, Lili Xu, Ying Chen, Yangang Wang

**Affiliations:** 1 Department of Endocrinology, Affiliated Hospital of Qingdao University, Qingdao, 266003 China; 2 Marine Biomedical Research Institute of Qingdao, School of Medicine and Pharmacy, Key Laboratory of Marine Drugs, Chinese Ministry of Education, Ocean University of China, Qingdao, 266003 China

**Keywords:** Joint association, DI-GM, Sleep disorders, Survival, NHANES

## Abstract

**Background:**

Diet and sleep disorders are associated with risks of metabolic diseases such as diabetes. The dietary index for gut microbiota (DI-GM) is a newly proposed index designed to assess dietary quality associated with maintaining a healthy gut microbiota. The authors aim to investigate the separate and joint prognostic effect of DI-GM and sleep disorders on the survival of US population with diabetes and pre-diabetes.

**Methods:**

Data were from the National Health and Nutrition Examination Survey (NHANES) 2007–2018 at baseline linked to the 2019 National Death Index records.

Dietary recall data were collected to calculate the DI-GM and sleep disorders were assessed by self-reported questionnaires. The Cox proportional hazard model were used to evaluate the associations between separate and joint prognostic effects of DI-GM and sleep disorders with mortality outcomes among diabetic and pre-diabetic patients.

**Results:**

A total of 10718 Participants with diabetes and pre-diabetes were ultimately included in this study (weighted population: 67,232,394, weighted mean age [SE]: 57.0 [0.1] years; weighted female proportion: 51.8%). Among these participants, higher DI-GM was more prevalent in those without sleep disorders. During the median follow-up of 13.3 years, 1448 deaths occurred, including 346 participants died from cancer, and 367 died from cardiovascular disease (CVD)..Multivariable models indicated that the joint effects of DI-GM (≥ 6) and no sleep disorders were associated with lower risks for all-cause (HR 0.53, 95% CI: 0.38–0.79) and CVD mortality (HR 0.36, 95% CI: 0.19–0.65).

**Conclusions:**

In a nationally representative sample of US population with diabetes and pre-diabetes, high DI-GM combined with no sleep disorders was associated with significantly reduced all-cause and CVD mortality risks.

**Supplementary Information:**

The online version contains supplementary material available at 10.1186/s12937-025-01162-0.

## Introduction

Diabetes is a significant public health problem worldwide, which is one of the leading causes of death in the United States. Epidemiological studies have demonstrated the association between diabetes and a variety of physical health conditions including cancer [1], cardiovascular disease (CVD) [2], renal diseases [3] as well as mortality from physical disorders. According to the latest statistics from the Centers for Disease Control and Prevention (CDC), as of 2021, approximately 97.6 million people aged 18 or older in the United States had pre-diabetes, accounting for 38.0% of the adult population [4]. Pre-diabetes may progress to diabetes, increasing the risk of CVD and death. Growing evidence indicates that disturbed sleep architecture is associated with impaired glucose homeostasis, decreased insulin secretion and an increased risk of diabetes [5, 6]. Recent studies have also found an association between poor subjective sleep and deterioration in blood glucose control [7]. Engeda et al. reported that pre-diabetes was associated with trouble maintaining sleep, waking up too early, and short sleep duration [8]. To our knowledge, the evidence regarding the impact of sleep disorders on the mortality risks of individuals with diabetes and pre-diabetes remains limited.

In recent years, an increasing number of studies have demonstrated that the gut microbiota and their metabolites play a crucial role in the development of diabetes through altering the intestinal mucosal barrier, influencing insulin secretion, and body immune regulation [9–11]. Healthy dietary patterns influence the composition and function of the gut microbiota [12]. Currently, one promising strategy for diabetes treatment is modulating gut microbiota composition by dietary interventions. The Mediterranean diet (MED), significantly alters the composition and function of the gut microbiota, increasing the production of short-chain fatty acids (SCFAs) [13]. The Dietary Approaches to Stop Hypertension (DASH) diet, with its high dietary fiber content [14],stimulates the growth of SCFA-producing bacteria, offering protective effects against diabetes [15]. SCFAs have been proved to enhance insulin sensitivity and reduce postprandial blood glucose levels [16]. A systematic review and dose–response meta-analysis found that high adherence to the DASH diet was significantly associated with a reduced risk of diabetes [17]. The MED intervention has been shown to increase the relative abundances of Lachnospiraceae NK4A136, which could improve patients'blood glucose levels, insulin levels, and insulin resistance [18]. With the deepening of research, recently, Kase and his colleagues developed a novel dietary index for gut microbiota (DI-GM) through a comprehensive literature review of longitudinal studies in order to measure the potential impact of diet on gut microbiota [19]. The DI-GM identified 14 foods or nutrients that have either beneficial or unfavorable effects on gut microbiota, with higher scores reflecting healthier gut microbiota. In addition, DI-GM was associated positively with markers of gut microbiota diversity biomarkers specifically urinary enterodiol and enterolactone, indicating that the dietary patterns captured by the DI-GM may contribute to a more diverse gut microbiota [19]. As a reliable tool, DI-GM can evaluate the impact of specific dietary modifications on the composition, and diversity of the gut microbial community, supporting microbiome-targeted dietary recommendations and personalized nutrition strategies for disease prevention and management. The specific impact of DI-GM on diabetes risk has not yet been thoroughly investigated.

Various evidence has shown that sleep has a complex interaction with microbial communities. The microbiota-gut-brain axis contributes to the regulation of sleep behavior both directly and indirectly and may play a critical role in the etiology and pathogenesis of sleep disorders. As a pathway for bidirectional brain-gut communication, the vagus nerve can regulate and receive information from the digestive system. Lactobacillus rhamnosus JB1 has been proved to ameliorate depression and anxiety through vagus nerve-dependent pathways and modulate GABA receptor expression in the brain [20]. Benzodiazepines targeting GABA receptors such as zolpidem are used to treat insomnia [21]. By modulating bacterial metabolites, neuronal signaling, endocrine signaling, and immune responses, the gut microbiota exerts an influence on sleep–wake behavior, in turn, lack of sleep leads to gut microbiota dysbiosis with changes in gut microbiome composition [1, 22]. Microbiota-targeted interventions may be promising novel strategies for themanagement of sleep disorders. To date, few studies have considered the influence of the joint effect of DI-GM and sleep disorders on the mortality risks of patients with diabetes and pre-diabetes. This study aimed to investigate the independent and joint associations of the newly proposed DI-GM and sleep disorders with all-cause, cancer, and CVD mortality among the US adult population with diabetes and pre-diabetes, utilizing National representative data from the NHANES Study. Our findings may bring benefits to the development of relevant evidence-based guidelines in patients with diabetes and pre-diabetes to improve their quality of life and minimize the risk of severe health outcomes.

## Methods

### Study design and population

**Fig. 1:**
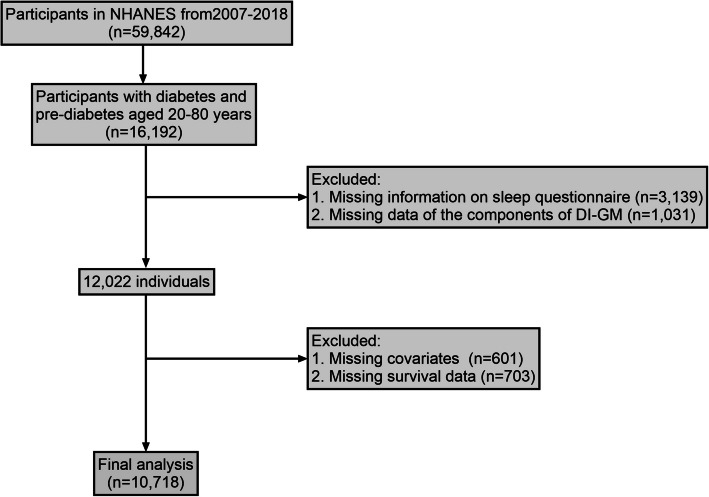
Flowchart of the study inclusion and exclusion of participants

This study utilized data collected from the National Health and Nutrition Examination Survey (NHANES), a series of cross-sectional, complex, multi-stage surveys, conducted by the Centers for Disease Control and Prevention (CDC) [23]. The NHANES study has been conducted on 2-year cycles since 1999 to monitor the health and nutritional status and diet of the US population. The protocol for the NHANES study was approved by the Institutional Review Board of the National Center for Health Statistics (NCHS), and written informed consent was provided by all participants. Participants in this study were invited to engage in a face-to-face interview, undergo a comprehensive series of physical examinations, and participate in laboratory tests in a mobile examination facility [24]. Six survey cycles of data in the NHANES database from 2007 to 2018 were analyzed in this study. Follow-up for mortality status was conducted for all individuals until the end of December 2019. Participants with missing data on glucose and HbA1c, cancer investigation, sleep questionnaire, dietary recall, and incomplete mortality follow-up data were excluded from this study. In total, 10718 participants with diabetes and pre-diabetes aged 20 years or older were eligible in the final analysis (Fig. 1).

### Diagnosis of diabetes and pre-diabetes

According to the American Diabetes Association (ADA) criteria [25], diabetes was diagnosed based on the presence of any of the following criteria: (a) fasting plasma glucose (FPG) level ≥ 126 mg/dL or HbA1c level ≥ 6.5%; (b) a response ‘yes’ to the question,"Has a doctor ever told you that you have diabetes?"or"Are you currently taking insulin or oral hypoglycemic medication?". Pre-diabetes was diagnosed based on the presence of any of the following criteria: (a) FPG level between 100 mg/dL and 125 mg/dL or HbA1c level between 5.7% and 6.4%; (b) a response ‘yes’ to the question,"Has a doctor ever told you that you have pre-diabetes?".

### Dietary index for gut microbiota

Kase et al. developed a novel dietary index for gut microbiota (DI-GM), which has been designed to reflect the dietary composition associated with the gut microbiota profiles [19]. They considered 14 foods or nutrients as integral components of the DI-GM including beneficial components like fermented dairy, chickpeas, soybean, fiber, cranberries, whole grains, avocados, broccoli, coffee and green tea, as well as unfavorable components like red meat, processed meat, refined grains, and high-fat diet (mainly soybean oil, ≥ 40% of energy from fat). Studies suggest high-fat diets are associated with reduced microbial abundance and increased gut permeability [26]. High-fat diets rich in meat protein may promote different and less diverse populations of sulphur-metabolising bacteria [27]. Thus high-fat diets act as unfavourable components of DI-GM. The components and scoring criteria for the DI-GM are detailed in the Supplementary Table S1. For beneficial to gut microbiota items, a value of 1 was assigned if the intake exceeded the sex-specific median, otherwise a value of 0 was given. For unfavorable to gut microbiota items, a value of 0 was assigned if the intake exceeded the sex-specific median or 40% (for High-fat diet), otherwise a value of 1 was given. The total DI-GM score ranges from 0 to 13, with beneficial items scoring from 0 to 9 and unfavorable items from 0 to 4. A higher score indicating a more favorable gut microbiota profile. In NHANES, The components of the DI-GM were derived from data obtained through two 24-h dietary recalls of all food and drink consumed on the day before the interview (from midnight to midnight), with a time interval varying from 3 to 10 days. The first dietary recall interview was collected face-to-face in the Mobile Examination Center (MEC) and the second interview was collected by telephone 3 to 10 days later. The mean intake of foods, food groups, and nutrients from the two 24-h recalls were used to construct the DI-GM. Previous studies have demonstrated that DI-GM score of ≥ 6 shows a significant negative correlation with adverse clinical outcomes, while the group scoring 0–3 is considered to be at high risk [28, 29]. In the present study, the DI-GM scores were categorized into three groups: 0–3, 4–6, and ≥ 6.

### Sleep disorders

The response to “Have you ever told a doctor or other health professional that you have trouble sleeping?” or “Have you ever been told by a doctor or other health professional that you have a sleep disorder?” in the Sleep Disorder Questionnaire, were used to assess sleep disorder and then participants were classified according to the answers (yes or no). Sleep time was categorized based on the question"How many hours of sleep do you usually get on weeknights or work nights?"The recorded sleep time was divided into short sleep (less than 7 h per night), normal sleep (7–9 h per night), and long sleep (more than 9 h per night).

### Ascertainment of mortality

The mortality data in this study were linked to the National Death Index (NDI) provided by NCHS, through December 31, 2019. The cause of death was coded by the 10th International Classification of Diseases (ICD-10). The study outcomes were all-cause mortality, cancer, and cardiovascular disease (CVD) mortality. The duration of follow-up was determined from the baseline interview to the date of death or December 31, 2019.

### Covariates

The selection of covariates was based on the knowledge regarding the factors influencing the prognosis of cancer survivors. Sociodemographic characteristics consisted of basic information, including age, gender(male or female), race/ethnicity(Mexican American, other Hispanic, non-Hispanic White, non-Hispanic Black, and other races), and educational level(Less Than 9th Grade, 9-11th Grade, High School Grad, Some College degree, College Graduate or above), family poverty income ratio (total family income divided by the poverty threshold; < 1.3, 1.3 to ≤ 3.5, > 3.5). Body mass index(BMI) was calculated by dividing weight (kg) by height in square meters and was classified as < 30, ≥ 30 kg/m^2^. Total calorie intake on the sum of two days (DR1TOT and DR2TOT) were utilized for analysis. Participants were considered non-smokers if they had either never smoked or had consumed fewer than 100 cigarettes in their lives. Smoking status was categorized, with"Yes"indicating participants who had smoked at least 100 cigarettes in their lives, and"No"for those who had either never smoked or had consumed fewer than 100 cigarettes. For alcohol use, the threshold for classifying"No"and"Yes"was based on having at least 12 alcoholic drinks per year. A history of hypertension and hypercholesterolemia was self-reported by participants who had been diagnosed by a physician or other health professional in a standardized medical condition questionnaire.

### Statistical analysis

Given the complex multistage stratified probability survey design employed by NHANES, all analyses in this study incorporated sample weights, clustering, and stratification to accurately estimate appropriate variance and ensure national representation of results. The baseline characteristics were presented according to DI-GM classification (0–3,4–6, ≥ 6), with the survey-weighted means and standard errors (SE) to describe continuous variables and weighted percentages with their 95% CI to describe categorical variables. In addition, to mitigate the impact of missing variables for covariates, we utilized the multiple imputation approach implemented in the"mice"package in R. Multivariable Cox proportional hazard regression model was utilized to determine the hazard ratios (HR) and their 95% CI for the associations of the DI-GM and sleep disorders with all-cause, cancer, and CVD mortality respectively. Fully adjusted models were adjusted for age, sex, race and ethnicity, educational attainment, family poverty income ratio, body mass index, total calorie intake, sleep time, smoking status, alcohol use, hypertension, and hypercholesterolemia. To explore the joint associations, participants were categorized based on the presence of the DI-GM and sleep disorders to assess mortality risks using multivariable Cox proportional hazards regression models with adjustments for the same set of covariates. All analyses were conducted in subpopulations according to age (< 65 years, ≥ 65 years), sex (male, female), and race and ethnicity (Mexican American, other Hispanic, non-Hispanic White, non-Hispanic Black and other race), using Cox proportional hazards regression models to examine the stability of the results. Finally, sensitivity analysis was performed by excluding participants who died within the initial 2-year follow-up, and had a history of cancer or CVD disease to lessen the probability of reverse causation.

All analyses were conducted using R version 4.4.1 and SPSS (IBM) version 27, and a two-sided *P* < 0.05 was set for a significant difference.

## Results

### Baseline characteristics

**Fig. 2:**
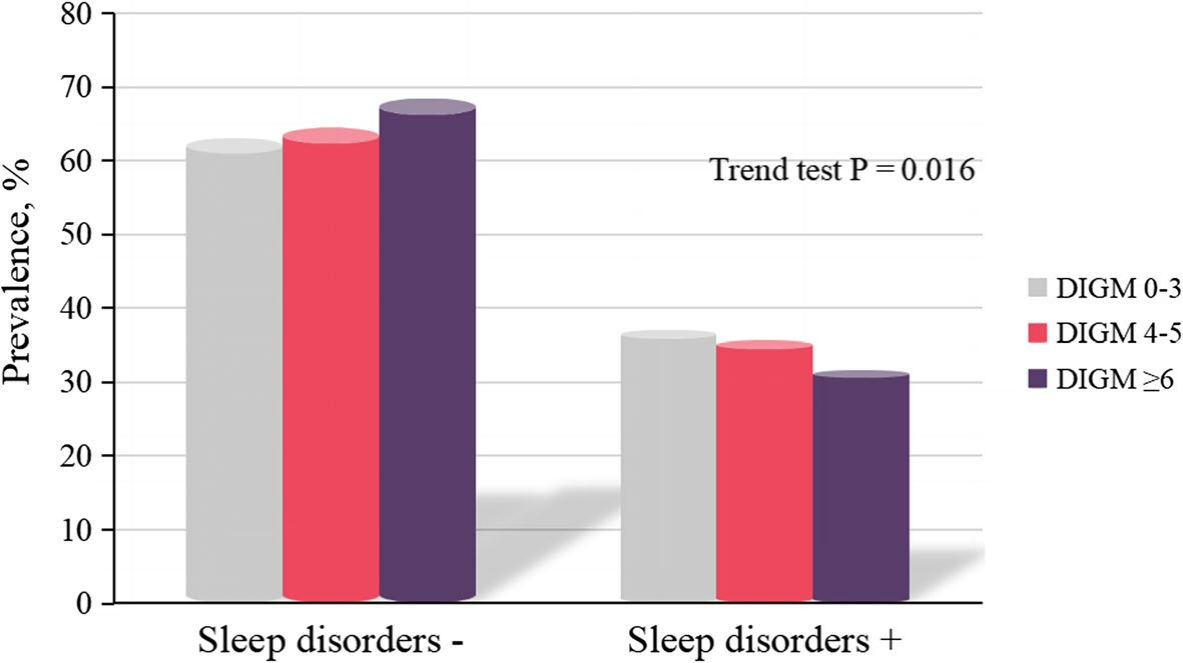
Joint prevalence of DI-GM and sleep disorders in a nationally representative sample of US population with diabetes and pre-diabetes, NHANES 2007 to 2018. Abbreviations: DI-GM, dietary index for gut microbiota; NHANES, National Health and Nutrition Examination Survey. Data were weighted to be nationally representative

A total of 10718 individuals with diabetes and pre-diabetes were ultimately included in this study (weighted population: 67,232,394, weighted mean age [SE]: 57.0 [0.1] years; weighted female proportion: 51.8%). The characteristics of the population categorized according to their DI-GM scores are presented in Table 1. Notably, participants with higher DI-GM (≥ 6) appeared to be more likely to be older, female, non-Hispanic White, have higher calories intake and normal sleep (7–9 h per night), and less likely to have hypertension and sleep disorders. The joint analysis of the prevalence of DI-GM and sleep disorders showed a significant downward trend in the prevalence of sleep disorders among participants with diabetes and pre-diabetes as DI-GM increased (Trend test *P* = 0.016) (Fig. 2).

**Table 1:** Baseline characteristics of US population stratified by groups of DI-GM, NHANES 2007 to 2018

Characteristics	Total	No. of participants by DI-GM group (weighted %)^a^		
		DI-GM 0–3	DI-GM 4–6	DI-GM ≥ 6
Overall	10718	1970 (17.4)	4958 (43.9)	3790 (38.7)
Age, mean (SE), year	57.0 (0.1)	56.3 (0.2)	56.2 (0.1)	58.9 (0.2)
Sex, n (%)				
Men	5279 (48.2)	1058 (52.7)	2462 (48.1)	1759 (46.1)
Women	5439 (51.8)	912 (47.3)	2496 (51.9)	2031 (53.9)
Race and ethnicity, n (%)				
Mexican American	1699 (9.0)	264 (8.1)	893 (0.8)	542 (7.3)
Other Hispanic	1161 (5.7)	210 (5.3)	558 (6.2)	393 (5.2)
Non-Hispanic White	3948 (51.8)	702 (51.3)	1692 (48.1)	1554 (56.1)
Non-Hispanic Black	2751 (25.0)	629 (29.6)	1330 (26.8)	792 (20.8)
Other Race	1159 (8.6)	165 (5.7)	485 (8.1)	509 (10.5)
Educational attainment, n (%)				
Less Than 9th Grade	1419 (13.2)	255 (13.0)	631 (13.2)	533 (13.4)
9-11th Grade	1678 (15.7)	312 (15.4)	753 (15.9)	613 (15.4)
High School Grad	2419 (22.7)	412 (21.0)	1158 (24.0)	849 (22.2)
Some College degree	2917 (27.0)	566 (28.1)	1343 (26.5)	1008 (26.7)
College Graduate or above	2285 (21.4)	425 (22.4)	1073 (20.4)	787 (22.3)
Family poverty income ratio, n (%)				
≤ 1.3	3709 (34.6)	688 (34.1)	1719 (34.5)	1302 (34.9)
> 1.3 to 3.5	3698 (33.7)	705 (35.7)	1674 (33.8)	1319 (32.7)
> 3.5	3311 (31.7)	577 (30.1)	1565 (31.7)	1169 (32.5)
BMI, kg/m^2^, n (%)				
< 30	7872 (73.7)	1436 (73.7)	3688 (75.0)	2748 (72.2)
≥ 30	2846 (26.3)	534 (26.3)	1270 (25.0)	1042 (27.8)
Total calories intake, mean (SE), kcal	3961.28 (15.0)	3903.85 (33.8)	3907.00 (22.9)	4048.65 (24.3)
Smoking status, n (%)				
No	5183 (49.2)	990 (48.5)	2409 (49.3)	1784 (49.4)
Yes	5535 (50.8)	980 (51.5)	2549 (5.7)	2006 (50.6)
Alcoholic use, n (%)				
No	4614 (71.2)	850 (73.6)	2130 (71.6)	1634 (69.7)
Yes	1808 (28.8)	311 (26.4)	834 (28.4)	663 (30.0)
Hypertension, n (%)				
No	7838 (75.2)	1428 (73.8)	3617 (74.8)	2793 (76.4)
Yes	2880 (24.8)	542 (26.2)	1341 (25.2)	997 (23.6)
Hyperlipidemia, n (%)				
No	5652(50.6)	1011 (50.8)	2596 (52.9)	2045 (55.0)
Yes	5067 (43.9)	960 (49.2)	2362 (47.1)	1745 (45.0)
Sleep disorders, n (%)				
No	7402 (65.7)	1287 (63.0)	3435 (64.4)	2680 (68.4)
Yes	3316 (34.3)	683 (37.0)	1523 (35.6)	1110 (31.6)
Sleep time, n (%)				
< 7	3838 (33.9)	709 (36.1)	1835 (35.1)	1294 (31.6)
7–9	6171 (60.1)	1089 (56.2)	2784 (58.8)	2298 (63.3)
> 9	709 (6.0)	172 (7.6)	339 (6.1)	198 (5.1)
DI-GM score, mean (SE)	4.95(0.02)	2.55(0.02)	4.51(0.01)	6.77(0.02)
Beneficial to gut microbiota, mean (SE)	2.29 (0.03)	0.97 (0.04)	1.85 (0.03)	3.56 (0.03)
Unfavorable to gut microbiota, mean (SE)	1.35 (0.01)	2.42 (0.02)	1.34 (0.01)	0.79 (0.01)
Diabetes, n (%)	4883 (41.3)	962 (46.7)	2310 (42.1)	1611 (38.1)
Pre-diabetes, n (%)	5835 (58.7)	1008 (53.3)	2648 (57.9)	2179 (61.9)

All means and SEs for continuous variables and percentages for categorical variables were weighted. The DI-GM score comprises BGMS and UGMS, categorized into three groups: 0–3, 4–5, and ≥ 6

*Abbreviations*: *BMI* body mass index (calculated as weight in kilograms divided by height in meters squared), *DI-GM* dietary index for gut microbiota, *h/d* hours per day, *NHANES* National Health and Nutrition Examination Survey, *SE* Standard Error

^a^The weighted percentages may not reach exactly 100% due to missing data

### Relationship between DI-GM, sleep disorders, and mortality

**Fig. 3:**
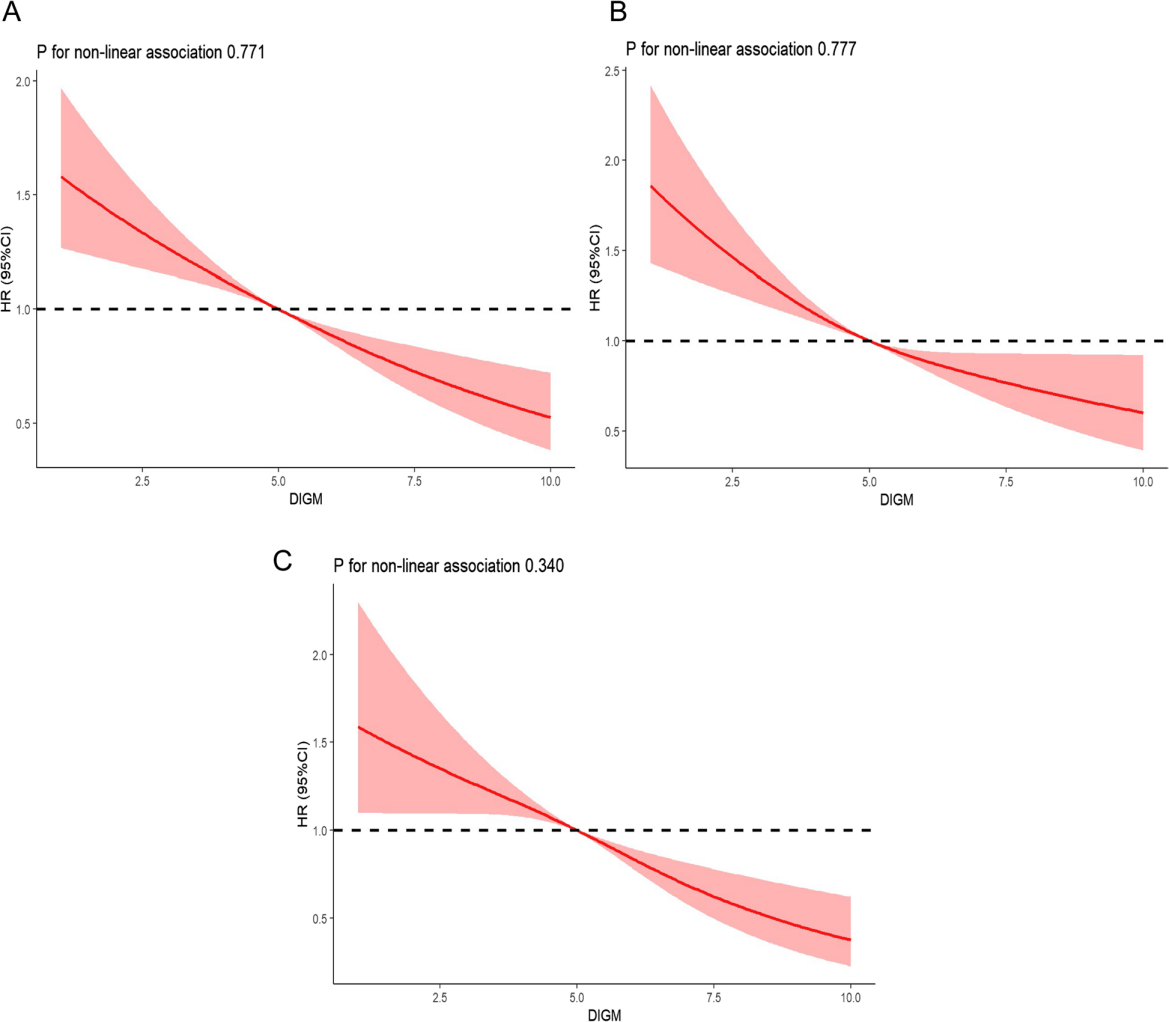
Dose–response association of DI-GM with all-cause mortality among US populations with diabetes and pre-diabetes (**A**), diabetes (**B**), and pre-diabetes (**C**), NHANES 2007 to 2018. The solid and dashed lines illustrate the estimated hazard ratios along with their respective 95% confidence intervals. Abbreviations: DI-GM, dietary index for gut microbiota; NHANES, the National Health and Nutrition Examination Survey. Adjusted for age, sex, race and ethnicity, educational attainment, family poverty income ratio, body mass index, total calories intake, sleep time, smoking status, alcohol use, hypertension, and hypercholesterolemia

During the follow-up period of up to 13.3 years (median, 6.4 years), 1448 deaths occurred, including 346 participants died from cancer, and 367 died from CVD. Participants with either high DI-GM (≥ 6) or no sleep disorders had reduced risks of all-cause and CVD mortality (Table 2). After adjusting for covariates, the HRs for all-cause, cancer, and CVD mortality among individuals with DI-GM (≥ 6) were 0.71 (95% CI: 0.57–0.93), 0.92 (95% CI: 0.70–1.54) and 0.63 (95% CI: 0.49–0.85), respectively, compared to those with DI-GM (0–3). Each 1-point increase in DI-GM was associated with 6% and 9% reduction in the risk of death from all-cause and CVD mortality, respectively. As shown in Fig. 3, we further considered diabetic and pre-diabetic individuals separately, finding that each 1-point increase in DI-GM was associated with 8% and 10% reduction in the risk of all-cause mortality, respectively. Additionally, the risks of all-cause and CVD mortality among individuals with diabetes and pre-diabetes decreased as the beneficial to gut microbiota increased (HR 0.84, 95% CI: 0.80–0.93; HR 0.81, 95% CI: 0.77–0.92), respectively, but the unfavorable to gut microbiota was not associated with an increased risk of mortality (Table 2). Meanwhile, the HRs for all-cause, cancer, and CVD mortality among individuals with sleep disorders were 1.28 (95% CI: 1.10–1.37), 1.10(95% CI: 0.82–1.47), and 1.44 (95% CI: 1.21–1.84), respectively, compared to those without sleep disorders.

**Table 2:** Association of DI-GM and sleep disorders with all-cause, cancer and CVD mortality among US population with diabetes and pre-diabetes, NHANES 2007 to 2018

	Mortality outcome							
Hazard ratio (95% CI), *P* value	All-cause mortality							
	DI-GM group						Sleep disorders	
	0–3	4–6	≥ 6	Per 1 point increase	Beneficial to gut microbiota	Unfavorable to gut microbiota	No	Yes
Death/No	311/1970	669/4958	468/3790	NA	NA	NA	954/7402	494/3316
Age adjusted^a^	Reference	0.86(0.70–1.06)	0.69(0.53–0.88)	0.90(0.85–0.95)	0.83(0.78–0.88)	1.05(0.98–1.13)	Reference	1.28(1.09–1.33)
P		0.165	< 0.001	< 0.001	< 0.001	0.153		< 0.001
MV model 1^b^	Reference	0.89(0.75–1.11)	0.71(0.57–0.92)	0.92(0.88–0.96)	0.83(0.79–0.91)	1.06(0.98–1.14)	Reference	1.27(1.09–1.35)
P		0.302	< 0.001	< 0.001	< 0.001	0.054		< 0.001
MV model 2^c^	Reference	0.91(0.76–1.13)	0.71(0.57–0.93)	0.94(0.86–0.97)	0.84(0.80–0.93)	1.08(1.02–1.19)	Reference	1.28(1.10–1.37)
P		0.315	< 0.001	< 0.001	< 0.001	0.073		< 0.001

	Mortality outcome							
Hazard ratio (95% CI), *P* value	Cancer mortality							
	DI-GM group						Sleep disorders	
	0–3	4–6	≥ 6	Per 1 point increase	Beneficial to gut microbiota	Unfavorable to gut microbiota	No	Yes
Death/No	70/1970	159/4958	111/3790	NA	NA	NA	234/7402	112/3316
Age adjusted^a^	Reference	0.93(0.70–1.54)	0.85(0.66–1.22)	0.92(0.86–1.02)	0.97(0.79–1.26)	1.04(0.74–1.39)	Reference	1.15(0.93–1.50)
P		0.682	0.236	0.097	0.136	0.523		0.279
MV model 1^b^	Reference	0.96(0.75–1.69)	0.89(0.70–1.39)	0.96(0.91–1.14)	0.97(0.80–1.29)	1.03(0.73–1.38)	Reference	1.11(0.86–1.48)
P		0.793	0.415	0.226	0.212	0.623		0.424
MV model 2^c^	Reference	0.98(0.77–1.74)	0.92(0.70–1.54)	0.97(0.91–1.18)	0.98(0.83–1.38)	1.03(0.71–1.39)	Reference	1.10(0.82–1.47)
P		0.902	0.589	0.297	0.447	0.764		0.472

	Mortality outcome							
Hazard ratio (95% CI), *P* value	CVD mortality							
	DI-GM group						Sleep disorders	
	0–3	4–6	≥ 6	Per 1 point increase	Beneficial to gut microbiota	Unfavorable to gut microbiota	No	Yes
Death/No	82/1970	176/4958	109/3790	NA	NA	NA	224/7402	143/3316
Age adjusted^a^	Reference	0.83(0.66–1.04)	0.61(0.47–0.79)	0.89(0.84–0.95)	0.81(0.76–0.86)	1.08(0.82–1.02)	Reference	1.39(1.11–1.78)
P		0.117	< 0.001	< 0.001	< 0.001	0.121		< 0.001
MV model 1^b^	Reference	0.85(0.68–1.08)	0.63(0.49–0.83)	0.90(0.85–0.96)	0.81(0.77–0.89)	1.10(0.98–1.20)	Reference	1.41(1.17–1.85)
P		0.179	< 0.001	< 0.001	< 0.001	0.053		< 0.001
MV model 2^c^	Reference	0.85(0.68–1.11)	0.63(0.49–0.85)	0.91(0.86–0.97)	0.81(0.77–0.92)	1.09(0.95–1.19)	Reference	1.44(1.21–1.84)
P		0.184	< 0.001	< 0.001	< 0.001	0.052		< 0.001

*Abbreviations*: *DI-GM* dietary index for gut microbiota, *NHANES* the National Health and Nutrition Examination Survey, *CI* confidence interval

^a^Adjusted for age

^b^Multivariable adjusted model additionally adjusted for sex, race and ethnicity, educational attainment, family poverty income ratio, body mass index, total calories intake, sleep time, smoking status, and alcohol use

^c^Additionally adjusted for hypertension, and hypercholesterolemia

^d^The DI-GM scores were categorized into three groups: 0–3, 4–6, and ≥ 6

### Joint association of DI-GM, sleep disorders with mortality

**Fig. 4:**
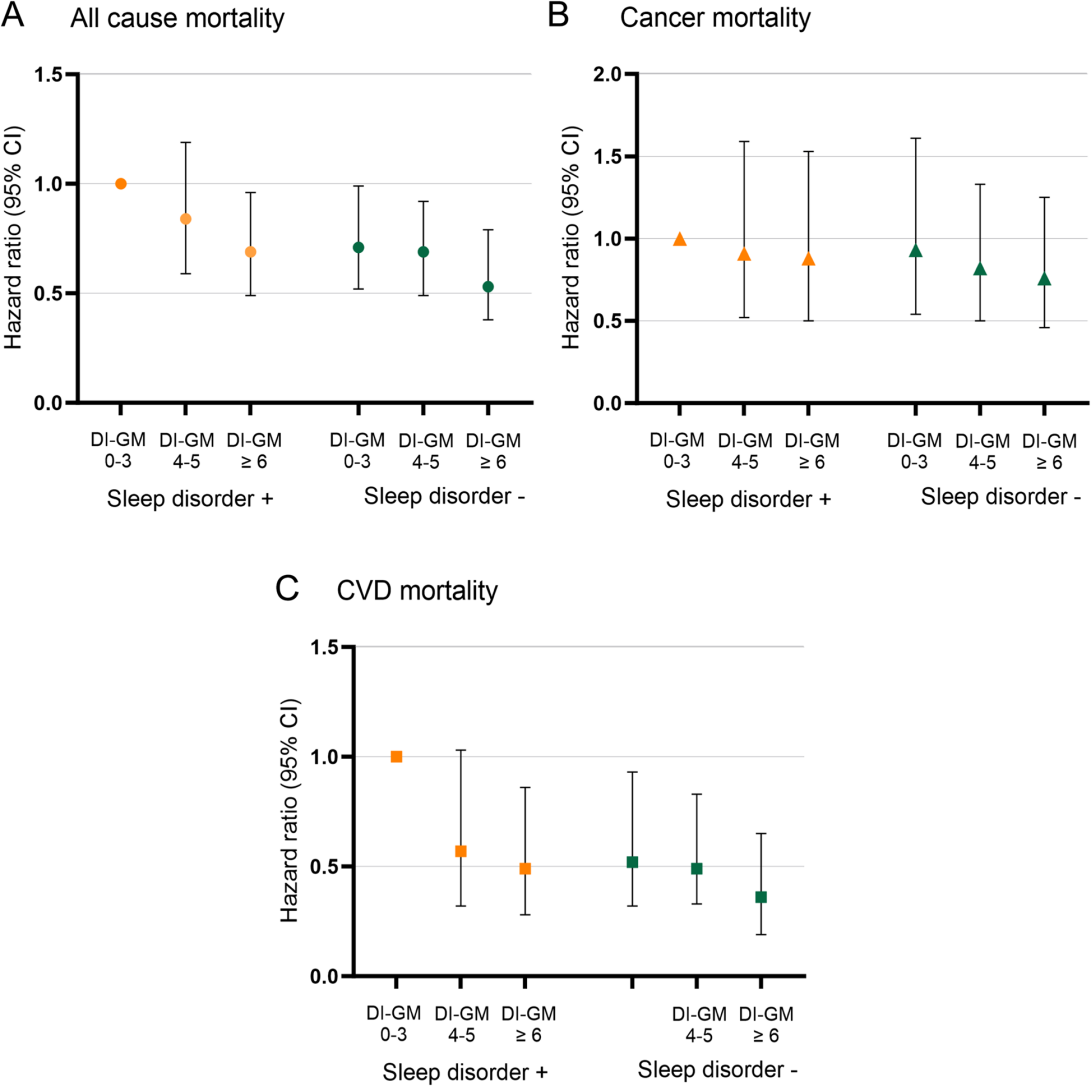
Joint association of DI-GM and sleep disorders with all-cause, cancer, and CVD mortality participants with diabetes and pre-diabetes, NHANES 2007 to 2018. Hazard ratios (solid symbols) with 95% CIs (error bars) of joint association between DI-GM and sleep disorders for all-cause (**A**), cancer (**B**), and CVD (**C**) mortality calculated using multivariable Cox regression models adjusted for age, sex, race and ethnicity, educational attainment, family poverty income ratio, body mass index, total calories intake, sleep time, smoking status, alcohol use, hypertension, and hypercholesterolemia. Abbreviations: DI-GM, dietary index for gut microbiota; NHANES, the National Health and Nutrition Examination Survey; CI, confidence interval; CVD, cardiovascular disease

In the joint analyses, diabetic and pre-diabetic participants with with DI-GM (≥ 6) and no sleep disorders had the lowest risks of all-cause and CVD mortality (Fig. 4). Specifically, compared with participants with DI-GM (0–3) and sleep disorders, the HRs for all-cause and CVD in the participants with DI-GM (≥ 6) and no sleep disorders were 0.53 (95% CI: 0.38–0.79) and 0.36 (95% CI: 0.19–0.65), respectively (Table 3). To ensure the reliability of our findings, a sensitivity analysis was performed. The main results remained similar after excluding the participants who died within 24 months(*n* = 303), participants with a history of cancer (*n* = 1269), and participants with a history of CVD (*n* = 964) (Supplementary Table S2-S4).

**Table 3:** Joint association of DI-GM and sleep disorders with all-cause, cancer, and CVD mortality among US population with diabetes and pre-diabetes, NHANES, 2007–2018

Mortality outcome	DI-GM group	Death/No	Hazard ratio (95% CI), *P* value					
			Age adjusted^a^		MV model 1^b^		MV model 2^c^	
All-cause mortality								
Sleep disorders	0–3	113/683	Reference		Reference		Reference	
	4–5	235/1523	0.83(0.58–1.17)	0.280	0.83(0.58–1.16)	0.255	0.84(0.59–1.19)	0.287
	≥ 6	146/1110	0.66(0.46–0.90)	< 0.001	0.69(0.48–0.93)	0.009	0.69(0.49–0.96)	0.012
No sleep disorders	0–3	198/1287	0.71(0.51–1.01)	0.054	0.70(0.51–0.99)	0.041	0.71(0.52–0.99)	0.048
	4–5	434/3435	0.65(0.48–0.85)	< 0.001	0.68(0.49–0.88)	< 0.001	0.69(0.49–0.92)	0.003
	≥ 6	322/2680	0.52(0.36–0.75)	< 0.001	0.54(0.39–0.75)	< 0.001	0.53(0.38–0.79)	< 0.001
P for trend				< 0.001		< 0.001		< 0.001
Cancer mortality								
Sleep disorders	0–3	25/683	Reference		Reference		Reference	
	4–5	52/1523	0.87(0.46–1.38)	0.306	0.86(0.50–1.46)	0.541	0.91(0.52–1.59)	0.742
	≥ 6	30/1110	0.74(0.44–1.24)	0.253	0.82(0.47–1.42)	0.472	0.88(0.50–1.53)	0.616
No sleep disorders	0–3	45/1287	0.91(0.50–1.56)	0.712	0.91(0.50–1.54)	0.870	0.93(0.54–1.61)	0.745
	4–5	107/3435	0.76(0.45–1.20)	0.240	0.83(0.51–1.35)	0.447	0.82(0.50–1.33)	0.413
	≥ 6	81/2680	0.65(0.41–1.03)	0.065	0.72(0.4–1.18)	0.193	0.76(0.46–1.25)	0.256
P for trend				0.114		0.207		0.258
CVD mortality								
Sleep disorders	0–3	37/683	Reference		Reference		Reference	
	4–5	69/1523	0.55(0.32–0.94)	0.030	0.56(0.34–0.97)	0.036	0.57(0.32–1.03)	0.054
	≥ 6	37/1110	0.47(0.26–0.84)	0.010	0.48(0.28–0.87)	0.014	0.49(0.28–0.86)	0.012
No sleep disorders	0–3	45/1287	0.48(0.27–0.87)	0.017	0.50(0.29–0.88)	0.017	0.52(0.32–0.93)	0.020
	4–5	107/3435	0.46(0.24–0.80)	< 0.001	0.48(0.28–0.81)	< 0.001	0.49(0.33–0.83)	< 0.001
	≥ 6	72/2680	0.34(0.19–0.62)	< 0.001	0.32(0.20–0.60)	< 0.001	0.36(0.19–0.65)	< 0.001
P for trend				< 0.001		< 0.001		< 0.001

*Abbreviations*: *DI-GM* dietary index for gut microbiota, *NHANES* the National Health and Nutrition Examination Survey, *CI* confidence interval, *CVD* cardiovascular disease

^a^Adjusted for age

^b^Multivariable adjusted model additionally adjusted for sex, race and ethnicity, educational attainment, family poverty income ratio, body mass index, total calories intake, sleep time, smoking status, and alcohol use

^c^Additionally adjusted for hypertension and hypercholesterolemia

### Subgroup analyses

In subgroup analyses, the results of the joint analysis of DI-GM and sleep disorders are detailed in Supplementary Table S5 and Supplementary Table S6. The joint effect of DI-GM and sleep disorders on all-cause mortality was significant in participants aged < 65 years (HR, 0.45; 95% CI, 0.23–0.81) and ≥ 65 years (HR, 0.59; 95% CI, 0.38–0.91), both male (HR, 0.54; 95% CI, 0.33–0.87) and female (HR, 0.51; 0.32–0.82) participants, participants with BMI < 30 (HR, 0.51; 95% CI, 0.34–0.79), Mexican–American participants (HR, 0.31; 95% CI, 0.12–0.85), other Hispanic participants (HR, 0.25; 95% CI, 0.11–0.57), non-Hispanic White participants (HR, 0.53; 95% CI, 0.34–0.81), and non-Hispanic Black participants (HR, 0.58; 95% CI, 0.35–0.94) (Supplementary Table S5). The association between DI-GM, sleep disorders, and CVD mortality was more pronounced in participants both aged < 65 years (HR, 0.28; 95% CI, 0.11–0.62) and ≥ 65 years (HR, 0.48; 95% CI, 0.28–0.82), both male (HR, 0.50; 95% CI, 0.28–0.89) and female (HR, 0.31; 0.17–0.57) participants, participants with both BMI < 30 (HR, 0.44; 95% CI, 0.27–0.73), and BMI ≥ 30 (HR, 0.29; 95% CI, 0.13–0.65), other Hispanic participants (HR, 0.23; 95% CI, 0.08–0.66), non-Hispanic Black participants (HR, 0.26; 95% CI, 0.11–0.64) and other race (HR, 0.13; 95% CI, 0.06–0.75)(Supplementary Table S6). Additionally, the combined impact of DI-GM and sleep disorders on all-cause mortality was similar in both diabetic and pre-diabetic participants. Notably, this joint effect was more strongly associated with CVD mortality in pre-diabetic participants (HR 0.32, 95% CI: 0.16–0.66), compared to those with diabetes (HR 0.49, 95% CI: 0.29–0.81) (Supplementary Table S6).

## Discussion

This study is the first to investigate the joint effect of DI-GM and sleep disorders on the mortality risks in a nationally representative US population with diabetes and pre-diabetes. Our results revealed that about 38.7% had DI-GM score of more than 6 points, and 30.9% had sleep disorders. Over the follow-up period of 13.3 years (median, 6.4 years), high DI-GM, increase in beneficial to gut microbiota, and no sleep disorders were associated with decreased risks of all-cause and CVD mortality. In the joint analysis, individuals with diabetes and pre-diabetes with DI-GM (0–3) and sleep disorders had a higher risk of all-cause and CVD mortality than those with a single risk factor. The significance of the association was consistently observed across sensitivity and subgroup analyses. The association between the joint effect of DI-GM and sleep disorders and CVD mortality was more pronounced in participants with pre-diabetes than diabetes. These findings underscore the potential of personalized dietary interventions focused on DI-GM coupled with sleep modulation for mortality risks prevention, particularly in diabetes and pre-diabetes population.

Previous studies have explored the relationship among sleep disorders, diabetes, and mortality [30–32]. A study by Schantz et al. [33] found that the presence of both diabetes and frequent sleep disturbances was associated with a higher risk of all-cause mortality compared to either condition alone. A large single-center cohort study reported that sleep duration was a significant indicator of all-cause, extended CVD, and non-extended CVD mortality among patients with diabetes, with the lowest risks observed for patients with 5–7 h of sleep [34]. Another cohort study of the UK Biobank discovered that healthy sleep patterns could reduce the negative impact of metabolic control on CVD death in people with diabetes [32]. People with diabetes and sleep disorders are susceptible to cardiac hypoxia, which worsens the severity of heart failure [35]. Our study further validated the detrimental impact of sleep disorders on the increased risk of all-cause and CVD mortality in individuals with diabetes independently, especially considering pre-diabetic patients. Similar results were not observed for cancer mortality. Laboratory studies in healthy adults have shown that sleep deprivation is associated with a 40% reduction in glucose clearance rate and increased sympathetic activity compared to a refreshed state, and the latter may deteriorate the status of insulin resistance [36, 37]. Sleep deprivation in people with diabetes is likely to worsen complications and interfere with blood glucose control and management, leading to an excess risk of mortality. Therefore, these findings indicate that sleep disorders could elevate the risk of mortality among patients with diabetes and prediabetes.

The influence of diet on the gut microbiota and its potential role in diabetes and pre-diabetes has been a significant focus of research. Fiber, defined beneficial component in DI-GM, has been reported to increase the proportion of beneficial gut microbes, and improve blood glucose homeostasis, serum metabolomics and systemic inflammation in patients with diabetes [38]. A high-fat diet, having unfavorable effects on gut microbiota within DI-GM, may contribute to diabetes through impaired glucose and lipid metabolism, which impairs the function of key metabolic organs such as the liver, pancreas, and adipose tissue [39]. The Mediterranean Diet (MED) is a plant-based eating pattern that includes a rich variety of vegetables, fruits, whole grains, legumes, and nuts, which are beneficial components in DI-GM. A study has shown that upgraded adherence to a MED pattern reduced the proportion of people with diabetes at high risk of CVD [40]. Recently, adults with pre-diabetes were randomly assigned to follow MED or gut microbiome modulates the effects of a personalized postprandial-targeting (PPT) diet in a 6-month dietary intervention. Studies indicated that the PPT diet induced more pronounced changes in the composition of the gut microbiome and improved glycemic control significantly more than a MED diet, which further affected cardiometabolic markers in pre-diabetes [41, 42]. Currently, available dietary indicators show inconsistent results in reflecting the diversity and abundance of the gut microbiota [43, 44]. DI-GM, as a newly proposed indicator, was designed to reflect the diversity of the gut microbiota and to measure diet quality about gut microbiome health purposely. Our study indicated that high DI-GM significantly reduced the risk of all-cause and CVD mortality in patients with diabetes and pre-diabetes. Based on a comprehensive dietary pattern rather than a single food or nutrient evaluation might provide stronger evidence of the rational dietary recommendation for mortality prevention.

The singular effects of diet or sleep disorders on the prevention of diabetes and pre-diabetes have been well-established, however, no studies have yet examined their joint effects on these conditions. Joint analysis allows us to provide more comprehensive survival guidance for patients with diabetes and pre-diabetes by exploring the unique and combined impact of each factor on mortality outcomes. Our results revealed that the combination of high DI-GM (≥ 6) and no sleep disorders could significantly decrease the risks of all-cause and CVD mortality among patients with diabetes and pre-diabetes as compared with either factor alone. According to our subgroup analyses, the stratification of the study population into two subgroups based on diabetes status did not significantly influence the primary outcomes. Notably, this joint effect was more strongly associated with CVD mortality in pre-diabetic participants compared to those with diabetes. The Da Qing Diabetes Study indicated that lifestyle interventions, including diet alone and diet plus exercise, over 6 years reduced the risk of CVD mortality and CVD events by about 30–50% in people with pre-diabetes over the subsequent 30 years [45]. The English National Health Service Diabetes Prevention Programme showed lifestyle intervention led to significant reductions in weight and HbA1c in pre-diabetic people [46]. These findings emphasize the importance of lifestyle interventions in preventing the risk of long-term death in patients with pre-diabetes, in which personalised gut–microbiota focused dietary interventions combined with sleep modulation may be a promising strategy to reduce the risk of death.

There are several possible biological explanations for our findings. The Brain-Gut-Microbiome Axis plays a central role in the pathophysiology of diabetes. Individual sleep status has an impact on metabolism by affecting the microbiota composition, which in turn can alter insulin metabolism and cognitive function. Sleep disorders cause proliferation of pathogenic bacteria, Gryllostomatidae and Enterobacteriaceae, as well as a decrease in bacteria producing SCFAs [47]. SCFAs stimulate the production and secretion of GLP-1, which directly promotes insulin secretion and inhibits glucagon secretion through interactions with pancreatic β cells [48]. Additionally, microbiota can also modulate the inflammatory response that leads to cognitive decline in a model of sleep deprivation (SD) [49]. Colonisation of the SD-associated microbiome induces gut leakage with higher circulation of LPS and other bacterial toxins. These microbial products and metabolites can activate inflammasomes, triggering systemic inflammation and impaired glucose metabolism [50]. Therefore, the DI-GM and sleep disorders have adverse biological effects on individuals with diabetes and pre-diabetes, inflammation and metabolic disorders may explain the combined effect of these two risk factors.

Our study has several strengths. To our knowledge, this is the first study to evaluate the joint effect of DI-GM and sleep disorders on mortality outcomes in a nationally representative sample of the population with diabetes and pre-diabetes. Sample weights, clustering and stratification were used to assess appropriate variance and ensure national representation of the US population. Additionally, the nationally representative sample included multiple races to increase generalisability to other populations with diabetes and pre-diabetes. Sensitivity and stratified analyses to evaluate the robustness of the main findings. The consistent findings after excluding the participants who died within 24 months or had a history of cancer or CVD add robustness to our main conclusions and reduce concerns about reverse causation.

Admittedly, some limitations should be noted in our study. Firstly, as a cross-sectional study, this research design cannot establish causal relationships between DI-GM and diabetes. Future prospective cohort studies and randomized controlled trials will be required to verify potential causality. Secondly, the data on dietary intake and sleep disorders were self-reported, and were subject to recall biases and misclassification. Thirdly, the selection of components in the DI-GM was constrained by the limited literature available on each type of food, while foods that have not been thoroughly investigated in relation to the gut microbiota were not included in the index. Finally, the type of sleep disorder was not clearly defined.

## Conclusion

In summary, this population-based cohort study reveals that the newly proposed DI-GM is negatively correlated with the prevalence of diabetes and pre-diabetes. Furthermore, diabetic and pre-diabetic individuals with high DI-GM and no sleep disorders are associated with significantly reduced all-cause and CVD mortality risks.

These results highlight the importance of considering both gut–microbiotafocused dietary interventions and sleep status in developing targeted intervention strategies to improve survival outcomes among diabetic and pre-diabetic individuals.

## Supplementary Information

Supplementary Material 1.

Supplementary Material 2.

Supplementary Material 3.

Supplementary Material 4.

Supplementary Material 5.

Supplementary Material 6.

## Data Availability

The data sets generated and analyzed during this study are available at NHANES website: https://www.cdc.gov/nchs/nhanes/index.htm.

## References

[1] Yang D, Huang W, Wu CW, et al. Acute sleep deprivation exacerbates systemic inflammation and psychiatry disorders through gut microbiota dysbiosis and disruption of circadian rhythms. Microbiol Res. 2023;268:127292. 36608535 10.1016/j.micres.2022.127292

[2] Kim MK, Lee KN, Han K, et al. Diabetes Duration, cholesterol levels, and risk of cardiovascular diseases in individuals with type 2 diabetes. J Clin Endocrinol Metab. 2024;109(12):e2317–23. 38366387 10.1210/clinem/dgae092 PMC11570539

[3] Shan S, Luo Z, Yao L, et al. Cross-country inequalities in disease burden and care quality of chronic kidney disease due to type 2 diabetes mellitus, 1990–2021: findings from the global burden of disease study 2021. Diabetes Obes Metab. 2024;26(12):5950–9. 39344843 10.1111/dom.15969

[4] National Diabetes Statistics Report. Prevention, Centers For Disease Control, 2023.

[5] Antza C, Kostopoulos G, Mostafa S, et al. The links between sleep duration, obesity and type 2 diabetes mellitus. J Endocrinol. 2022;252(2):125–41. 10.1530/JOE-21-0155 PMC8679843 34779405

[6] Mao Y. Sleep architecture changes in diabetes. J Clin Med. 2024;13(22):6851. 39597994 10.3390/jcm13226851 PMC11594902

[7] Zhang Y, Liu C, Xu Y, et al. The relationship between sugar-sweetened beverages, sleep disorders, and diabesity. Front Endocrinol. 2023;13:1041977. 10.3389/fendo.2022.1041977 PMC9869278 36699031

[8] Engeda J, Mezuk B, Ratliff S, et al. Association between duration and quality of sleep and the risk of pre-diabetes: evidence fromNHANES. Diabet Med. 2013;30(6):676–80. 23425048 10.1111/dme.12165 PMC3660430

[9] Pöysti S, Silojärvi S, Brodnicki TC, et al. Gut dysbiosis promotes islet-autoimmunity by increasing T-cell attraction in islets via CXCL10 chemokine[J]. J Autoimmun. 2023;140:103090. 37572540 10.1016/j.jaut.2023.103090

[10] Yuan M, Sun T, Zhang Y, et al. Quercetin alleviates insulin resistance and repairs intestinal barrier in db/db mice by modulating gut microbiota. Nutrients. 2024;16(12):1870. 38931226 10.3390/nu16121870 PMC11206920

[11] Rosell-Mases E, Santiago A, Corral-Pujol M, et al. Mutual modulation of gut microbiota and the immune system in type 1 diabetes models. Nat Commun. 2023;14(1):7770. 38012160 10.1038/s41467-023-43652-x PMC10682479

[12] Dong H, Zhuang H, Yu C, et al. Interactions between soluble dietary fibers from three edible fungi and gut microbiota. Int J Biol Macromol. 2024;278(Pt 3):134685. 39168729 10.1016/j.ijbiomac.2024.134685

[13] Ross FC, Patangia D, Grimaud G, et al. The interplay between diet and the gut microbiome: implications for health and disease. Nat Rev Microbiol. 2024;22(11):671–86. 39009882 10.1038/s41579-024-01068-4

[14] Toi PL, Anothaisintawee T, Chaikledkaew U, et al. Preventive role of diet interventions and dietary factors in type 2 diabetes mellitus: an umbrella review. Nutrients. 2020;12(9):2722. 32899917 10.3390/nu12092722 PMC7551929

[15] Li YJ, Chen X, Kwan TK, et al. Dietary fiber protects against diabetic nephropathy through short-chain fatty acid-mediated activation of G protein-coupled receptors GPR43 and GPR109A. J Am Soc Nephrol. 2020;31(6):1267–81. 32358041 10.1681/ASN.2019101029 PMC7269358

[16] Vitale M, Giacco R, Laiola M, et al. Acute and chronic improvement in postprandial glucose metabolism by a diet resembling the traditional Mediterranean dietary pattern: Can SCFAs play a role? Clin Nutr. 2021;40(2):428–37. 32698959 10.1016/j.clnu.2020.05.025

[17] Quan X, Shen X, Li C, et al. Adherence to the dietary approaches to stop hypertension diet reduces the risk of diabetes mellitus: a systematic review and dose-response meta-analysis. Endocrine. 2024;86(1):85–100. 38816664 10.1007/s12020-024-03882-5 PMC11445359

[18] Galié S, García Gavilán J, Camacho Barcía L, et al. Effects of the mediterranean diet or nut consumption on gut microbiota composition and fecal metabolites and their relationship with cardiometabolic risk factors. Mol Nutr Food Res. 2021;65(19):e2000982. 34331403 10.1002/mnfr.202000982

[19] Kase BE, Liese AD, Zhang J, et al. The development and evaluation of a literature-based dietary index for gut microbiota. Nutrients. 2024;16(7):1045. 38613077 10.3390/nu16071045 PMC11013161

[20] Bravo JA, Forsythe P, Chew MV, et al. Ingestion of Lactobacillus strain regulates emotional behavior and central GABA receptor expression in a mouse via the vagus nerve. Proc Natl Acad Sci U S A. 2011;108(38):16050–5. 21876150 10.1073/pnas.1102999108 PMC3179073

[21] Nutt DJ, Stahl SM. Searching for perfect sleep: the continuing evolution of GABAA receptor modulators as hypnotics. J Psychopharmacol. 2010;24(11):1601–12. 19942638 10.1177/0269881109106927

[22] Wang X, Wang Z, Cao J, et al. Gut microbiota-derived metabolites mediate the neuroprotective effect of melatonin in cognitive impairment induced by sleep deprivation. Microbiome. 2023;11(1):17. 36721179 10.1186/s40168-022-01452-3 PMC9887785

[23] National Center for Health Statistics. National Health and Nutrition Examination Survey, 2015.

[24] Zipf G, Chiappa M, Porter KS, et al. National health and nutrition examination survey: plan and operations, 1999-2010. Vital Health Stat 1. 2013;56:1–37. 25078429

[25] ElSayed NA, Aleppo G, Aroda VR, et al. 2. Classification and diagnosis of diabetes:standards of care in diabetes—2023. Diabetes Care. 2023;46(Supplement_1):S19–40. 36507649 10.2337/dc23-S002 PMC9810477

[26] Malesza IJ, Malesza M, Walkowiak J, et al. High-fat, Western-style diet, systemic inflammation, and gut microbiota: a narrative review. Cells. 2021;10(11):3164. 34831387 10.3390/cells10113164 PMC8619527

[27] Nguyen LH, Ma W, Wang DD, et al. Association between sulfur-metabolizing bacterial communities in stool and risk of distal colorectal cancer in men. Gastroenterology. 2020;158(5):1313–25. 31972239 10.1053/j.gastro.2019.12.029 PMC7384232

[28] Liu J, Huang S. Dietary index for gut microbiota is associated with stroke among US adults[J]. Food Funct. 2025;16(4):1458–68. 39898733 10.1039/d4fo04649h

[29] Zhang X, Yang Q, Huang J, et al. Association of the newly proposed dietary index for gut microbiota and depression: the mediation effect of phenotypic age and body mass index. Eur Arch Psychiatry Clin Neurosci, 2024. 10.1007/s00406-024-01912-x 39375215

[30] Wang Y, Huang W, O Neil A, et al. Association between sleep duration and mortality risk among adults with type 2 diabetes: a prospective cohort study. Diabetologia. 2020;63(11):2292–304. 32671413 10.1007/s00125-020-05214-4 PMC7527363

[31] Gu K, Min SH, Cho J. Sleep duration and mortality in patients with diabetes: results from the 2007–2015 Korea national health and nutrition examination survey. Diabetes Metab. 2022;48(3):101312. 34896596 10.1016/j.diabet.2021.101312

[32] Li J, Yin J, Luo Y, et al. Association of healthy sleep pattern with the risk of cardiovascular disease and all-cause mortality among people with diabetes: a prospective cohort study. Diabetes Res Clin Pract. 2022;186:109822. 35271877 10.1016/j.diabres.2022.109822

[33] von Schantz M, Ong JC, Knutson KL. Associations between sleep disturbances, diabetes and mortality in the UK Biobank cohort: a prospective population-based study. J Sleep Res. 2021;30(6):e13392. 34101927 10.1111/jsr.13392 PMC8612946

[34] Li C, Lin C, Liu C, et al. Sleep duration predicts subsequent long-term mortality in patients with type 2 diabetes: a large single-center cohort study. Cardiovasc Diabetol. 2022;21(1):60. 35477572 10.1186/s12933-022-01500-0 PMC9045470

[35] Mansor LS, Mehta K, Aksentijevic D, et al. Increased oxidative metabolism following hypoxia in the type 2 diabetic heart, despite normal hypoxia signalling and metabolic adaptation. J Physiol. 2016;594(2):307–20. 26574233 10.1113/JP271242 PMC4713751

[36] Spiegel K, Leproult R, Van Cauter E. Impact of sleep debt on metabolic and endocrine function. The Lancet. 1999;354(9188):1435–9. 10.1016/S0140-6736(99)01376-8 10543671

[37] Sakamoto K, Butera MA, Zhou C, et al. Overnutrition causes insulin resistance and metabolic disorder through increased sympathetic nervous system activity. Cell Metab. 2025;37(1):121–37. 39437790 10.1016/j.cmet.2024.09.012 PMC11711004

[38] Chen L, Liu B, Ren L, et al. High-fiber diet ameliorates gut microbiota, serum metabolism and emotional mood in type 2 diabetes patients. Front Cell Infect Microbiol. 2023;13:1069954. 36794003 10.3389/fcimb.2023.1069954 PMC9922700

[39] Prasad M, Rajagopal P, Devarajan N, et al. A comprehensive review on high -fat diet-induced diabetes mellitus: an epigenetic view. J Nutr Biochem. 2022;107:109037. 35533900 10.1016/j.jnutbio.2022.109037

[40] Martínez-González MA, Montero P, Ruiz-Canela M, et al. Yearly attained adherence to Mediterranean diet and incidence of diabetes in a large randomized trial. Cardiovasc Diabetol. 2023;22(1):262. 37775736 10.1186/s12933-023-01994-2 PMC10542699

[41] Ben-Yacov O, Godneva A, Rein M, et al. Gut microbiome modulates the effects of a personalised postprandial-targeting (PPT) diet on cardiometabolic markers: a diet intervention in pre-diabetes. Gut. 2023;72(8):1486–96. 37137684 10.1136/gutjnl-2022-329201 PMC10359530

[42] Ben-Yacov O, Godneva A, Rein M, et al. Personalized postprandial glucose response-targeting diet versus mediterranean diet for glycemic control in prediabetes. Diabetes Care. 2021;44(9):1980–91. 34301736 10.2337/dc21-0162

[43] Cotillard A, Cartier-Meheust A, Litwin NS, et al. A posteriori dietary patterns better explain variations of the gut microbiome than individual markers in the American Gut Project. Am J Clin Nutr. 2022;115(2):432–43. 34617562 10.1093/ajcn/nqab332 PMC8827078

[44] Maskarinec G, Hullar MAJ, Monroe KR, et al. Fecal microbial diversity and structure are associated with diet quality in the multiethnic cohort adiposity phenotype study. J Nutr. 2019;149(9):1575–84. 31187868 10.1093/jn/nxz065 PMC6862930

[45] Yu L, Wang J, Gong Q, et al. Influence of a diet and/or exercise intervention on long-term mortality and vascular complications in people with impaired glucose tolerance: da qing diabetes prevention outcome study. Diabetes Obes Metab. 2024;26(4):1188–96. 38168886 10.1111/dom.15413

[46] Valabhji J, Barron E, Bradley D, et al. Early outcomes from the english national health service diabetes prevention programme. Diabetes Care. 2020;43(1):152–60. 31719054 10.2337/dc19-1425 PMC7115827

[47] Li Y, Shao L, Mou Y, et al. Sleep, circadian rhythm and gut microbiota: alterations in Alzheimer’s disease and their potential links in the pathogenesis. Gut Microbes. 2021;13(1):1957407. 34520319 10.1080/19490976.2021.1957407 PMC8463034

[48] Zhao L, Zhang F, Ding X, et al. Gut bacteria selectively promoted by dietary fibers alleviate type 2 diabetes. Science. 2018;359(6380):1151–6. 29590046 10.1126/science.aao5774

[49] Wang Z, Chen WH, Li SX, et al. Gut microbiota modulates the inflammatory response and cognitive impairment induced by sleep deprivation. Mol Psychiatry. 2021;26(11):6277–92. 33963281 10.1038/s41380-021-01113-1

[50] Guo Z, Pan J, Zhu H, et al. Metabolites of gut microbiota and possible implication in development of diabetes mellitus. J Agric Food Chem. 2022;70(20):5945–60. 35549332 10.1021/acs.jafc.1c07851

