# Supplementary material for: Joint association of the newly proposed dietary index for gut microbiota and sleep disorders with survival among US adult population with diabetes and pre-diabetes
Source: Nutr J. 2025 Jun 18;24:95. doi: 10.1186/s12937-025-01162-0 (PMC12175418; doi:10.1186/s12937-025-01162-0)
Supplement: Supplementary file 1 — Supplementary Material 1. [file 12937_2025_1162_MOESM1_ESM.docx]

**Supplementary Table S1**

The components of DI-GM and scoring criteriain the US National Health and Nutrition Examination Survey (NHANES).

| **Components** | **Scoring criteria** | |
| --- | --- | --- |
| **Beneficial to gut microbiota** | 1 | 0 |
| Avocados | Consumption≥sex-specific median | Consumption<sex-specific median |
| Broccoli |  |  |
| Chickpeas |  |  |
| Coffee |  |  |
| Cranberries |  |  |
| Fermented dairy  (including yogurt, cheese, kefir, sour cream, buttermilk) |  |  |
| Fiber |  |  |
| Soybean  (including Soy milk, Tofu) |  |  |
| Whole grains |  |  |
| **Unfavorable to gut microbiota** |  |  |
| Processed meat  (Frankfurters, sausages, corned beef, and luncheon meat that are made from beef, pork, or poultry) | Consumption<sex-specific median | Consumption≥sex-specific median |
| Red meat  (Beef, veal, pork, lamb, and game meat; excludes organ meat and cured meat) |  |  |
| Refined grains  (Refined grains that do not contain all of the components of the entire grain kernel) |  |  |
| High-fat diet (% energy) | Consumption<40% | Consumption≥40% |
