# Supplementary material for: Joint association of the newly proposed dietary index for gut microbiota and sleep disorders with survival among US adult population with diabetes and pre-diabetes
Source: Nutr J. 2025 Jun 18;24:95. doi: 10.1186/s12937-025-01162-0 (PMC12175418; doi:10.1186/s12937-025-01162-0)
Supplement: Supplementary file 2 — Supplementary Material 2. [file 12937_2025_1162_MOESM2_ESM.docx]

**Supplementary Table S2**

Sensitive analysis of the joint association of DI-GM and sleep disorders with the all-cause mortality.

| **Mortality outcome** | **DI-GM group** | **Hazard ratio (95% CI)** |
| --- | --- | --- |
| **Exclusion of deaths during the first two years of follow-up** | |  |
|  | |  |
| Sleep disorders | 0-3 | Reference |
|  | 4-5 | 0.80 (0.54-1.18) |
|  | ≥6 | 0.74 (0.51-1.08) |
| No sleep disorders | 0-3 | 0.76 (0.49-1.16) |
|  | 4-5 | 0.73 (0.51-0.93) |
|  | ≥6 | 0.58 (0.38-0.76) |
| **P for trend** | | ＜0.001 |
| **Exclusion of deaths with history of cancers** | |  |
| Sleep disorders | 0-3 | Reference |
|  | 4-5 | 0.75 (0.49-1.14) |
|  | ≥6 | 0.64 (0.41-0.98) |
| No sleep disorders | 0-3 | 0.69 (0.48-0.98) |
|  | 4-5 | 0.65 (0.45-0.97) |
|  | ≥6 | 0.50 (0.35-0.75) |
| **P for trend** | | ＜0.001 |
| **Exclusion of deaths with history of CVD** | |  |
| Sleep disorders | 0-3 | Reference |
|  | 4-5 | 0.82 (0.55-1.22) |
|  | ≥6 | 0.76 (0.51-1.12) |
| No sleep disorders | 0-3 | 0.67 (0.47-0.95) |
|  | 4-5 | 0.63 (0.42-0.94) |
|  | ≥6 | 0.57 (0.38-0.85) |
| **P for trend** | | ＜0.001 |
