# Supplementary material for: Joint association of the newly proposed dietary index for gut microbiota and sleep disorders with survival among US adult population with diabetes and pre-diabetes
Source: Nutr J. 2025 Jun 18;24:95. doi: 10.1186/s12937-025-01162-0 (PMC12175418; doi:10.1186/s12937-025-01162-0)
Supplement: Supplementary file 3 — Supplementary Material 3. [file 12937_2025_1162_MOESM3_ESM.docx]

**Supplementary Table S3**

Sensitive analysis of the joint association of DI-GM and sleep disorders with cancer mortality.

| **Mortality outcome** | **DI-GM group** | **Hazard ratio (95% CI)** |
| --- | --- | --- |
| **Exclusion of deaths during the first two years of follow-up** | |  |
|  | |  |
| Sleep disorders | 0-3 | Reference |
|  | 4-5 | 0.94 (0.51-1.77) |
|  | ≥6 | 0.83 (0.44-1.56) |
| No sleep disorders | 0-3 | 1.05 (0.57-1.95) |
|  | 4-5 | 0.83 (0.47-1.47) |
|  | ≥6 | 0.79 (0.41-1.54) |
| **P for trend** | | 0.501 |
| **Exclusion of deaths with history of cancers** | |  |
| Sleep disorders | 0-3 | Reference |
|  | 4-5 | 0.93 (0.51-1.68) |
|  | ≥6 | 0.84 (0.43-1.66) |
| No sleep disorders | 0-3 | 1.02 (0.54-1.94) |
|  | 4-5 | 0.84 (0.47-1.56) |
|  | ≥6 | 0.77 (0.40-1.51) |
| **P for trend** | | 0.487 |
| **Exclusion of deaths with history of CVD** | |  |
| Sleep disorders | 0-3 | Reference |
|  | 4-5 | 0.89 (0.49-1.61) |
|  | ≥6 | 0.78 (0.43-1.42) |
| No sleep disorders | 0-3 | 0.94 (0.53-1.67) |
|  | 4-5 | 0.84 (0.48-1.48) |
|  | ≥6 | 0.81 (0.45-1.36) |
| **P for trend** | | 0.665 |
