# Supplementary material for: Joint association of the newly proposed dietary index for gut microbiota and sleep disorders with survival among US adult population with diabetes and pre-diabetes
Source: Nutr J. 2025 Jun 18;24:95. doi: 10.1186/s12937-025-01162-0 (PMC12175418; doi:10.1186/s12937-025-01162-0)
Supplement: Supplementary file 4 — Supplementary Material 4. [file 12937_2025_1162_MOESM4_ESM.docx]

**Supplementary Table S4**

Sensitive analysis of the joint association of DI-GM and sleep disorders with CVD mortality.

| **Mortality outcome** | **DI-GM group** | **Hazard ratio (95% CI)** |
| --- | --- | --- |
| **Exclusion of deaths during the first two years of follow-up** | |  |
|  | |  |
| Sleep disorders | 0-3 | Reference |
|  | 4-5 | 0.56 (0.32-0.97) |
|  | ≥6 | 0.49 (0.28-0.85) |
| No sleep disorders | 0-3 | 0.51 (0.30-0.86) |
|  | 4-5 | 0.42 (0.22-0.80) |
|  | ≥6 | 0.33 (0.18-0.60) |
| **P for trend** | | ＜0.001 |
| **Exclusion of deaths with history of cancers** | |  |
| Sleep disorders | 0-3 | Reference |
|  | 4-5 | 0.56 (0.32-1.01) |
|  | ≥6 | 0.42 (0.23-0.74) |
| No sleep disorders | 0-3 | 0.52 (0.30-0.87) |
|  | 4-5 | 0.40 (0.21-0.75) |
|  | ≥6 | 0.29 (0.14-0.58) |
| **P for trend** | | ＜0.001 |
| **Exclusion of deaths with history of CVD** | |  |
| Sleep disorders | 0-3 | Reference |
|  | 4-5 | 0.61 (0.33-1.03) |
|  | ≥6 | 0.43 (0.24-0.77) |
| No sleep disorders | 0-3 | 0.54 (0.27-0.94) |
|  | 4-5 | 0.35(0.18-0.57) |
|  | ≥6 | 0.32 (0.16-0.53) |
| **P for trend** | | ＜0.001 |
