# Supplementary material for: Joint association of the newly proposed dietary index for gut microbiota and sleep disorders with survival among US adult population with diabetes and pre-diabetes
Source: Nutr J. 2025 Jun 18;24:95. doi: 10.1186/s12937-025-01162-0 (PMC12175418; doi:10.1186/s12937-025-01162-0)
Supplement: Supplementary file 5 — Supplementary Material 5. [file 12937_2025_1162_MOESM5_ESM.docx]

**Supplementary Table S5**

The subgroup analysis of joint association of DI-GM and sleep disorders with all-cause mortality among US population with diabetes and pre-diabetes.

|  | **Death/No.** | |  | **All-cause mortality** |  |
| --- | --- | --- | --- | --- | --- |
|  | DI-GM 0-3/ sleep disorders | DI-GM ≥6 /no sleep disorders | HR (95% CI) |  | P value |
| All patients | 113/683 | 322/2680 | 0.53 (0.37-0.76 | 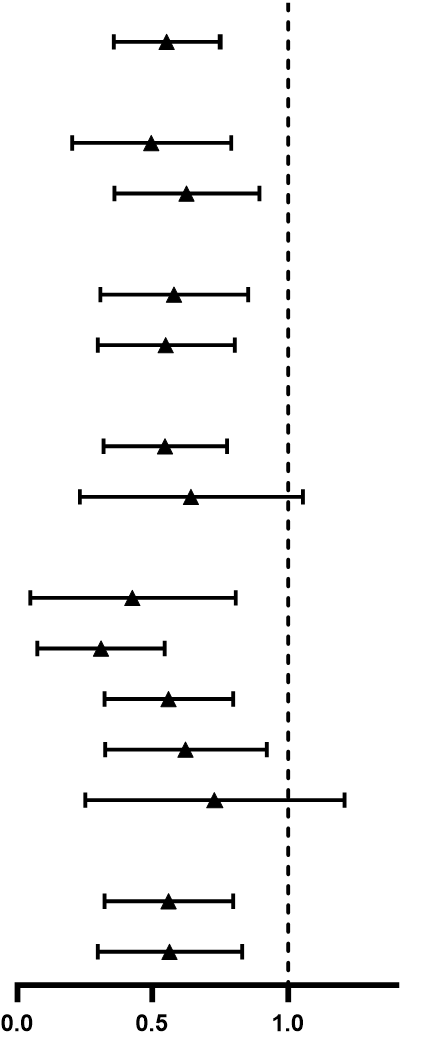 | <0.001 |
| Age, y |  |  |  |  |  |
| <65 | 46/434 | 69/1543 | 0.45 (0.23–0.81) |  | 0.006 |
| ≥65 | 67/249 | 253/1137 | 0.59 (0.38–0.91) |  | 0.018 |
| Sex |  |  |  |  |  |
| Male | 60/330 | 179/1321 | 0.54 (0.33–0.87) |  | 0.004 |
| Female | 53/353 | 143/1359 | 0.51 (0.32-0.82) |  | <0.001 |
| BMI, kg/m2 |  |  |  |  |  |
| <30 | 85/506 | 226/1945 | 0.51 (0.34-0.79) |  | 0.002 |
| ≥30 | 28/177 | 96/735 | 0.56 (0.28-1.09) |  | 0.089 |
| Race and ethnicity |  |  |  |  |  |
| Mexican American | 7/61 | 27/410 | 0.31 (0.12-0.85) |  | 0.012 |
| Other Hispanic | 10/63 | 20/283 | 0.25 (0.11–0.57) |  | <0.001 |
| Non-Hispanic White | 68/285 | 207-205/1033 | 0.53 (0.34-0.81) |  | 0.003 |
| Non-Hispanic Black | 26/224 | 56-54/552 | 0.58 (0.35-0.94) |  | 0.027 |
| Other Race | 2/45 | 12-16/402 | 0.60 (0.33–1.26) |  | 0.210 |
| Diabetes status |  |  |  |  | 0.003 |
| Diabetes | 78/379 | 175/1054 | 0.53 (0.34-0.81) |  | 0.012 |
| Pre-diabetes | 35/304 | 147/1626 | 0.54 (0.31-0.84) |  | 0.023 |
|  |  |  |  |  |  |

Adjusted hazard ratios for all-cause mortality in participants with DI-GM ≥6 and no sleep disorders, compared to participants with DI-GM 0-3 and sleep disorders, stratified by age, sex, BMI, and race and ethnicity and diabetes status.
