# Supplementary material for: Joint association of the newly proposed dietary index for gut microbiota and sleep disorders with survival among US adult population with diabetes and pre-diabetes
Source: Nutr J. 2025 Jun 18;24:95. doi: 10.1186/s12937-025-01162-0 (PMC12175418; doi:10.1186/s12937-025-01162-0)
Supplement: Supplementary file 6 — Supplementary Material 6. [file 12937_2025_1162_MOESM6_ESM.docx]

Supplementary Table S6

The subgroup analysis of joint association of DI-GM and sleep disorders with cancer and CVD mortality among US population with diabetes and pre-diabetes.

| Subgroup | HR (95% CI) | 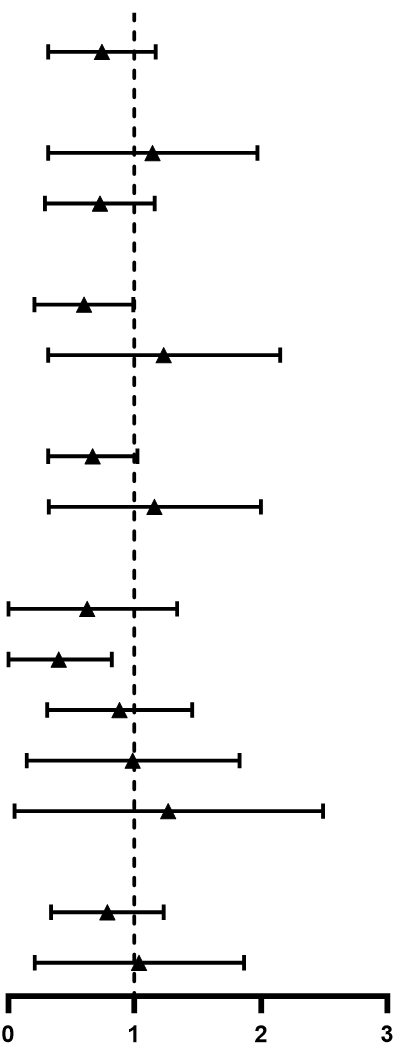**Cancer mortality** | P value | HR (95% CI) | 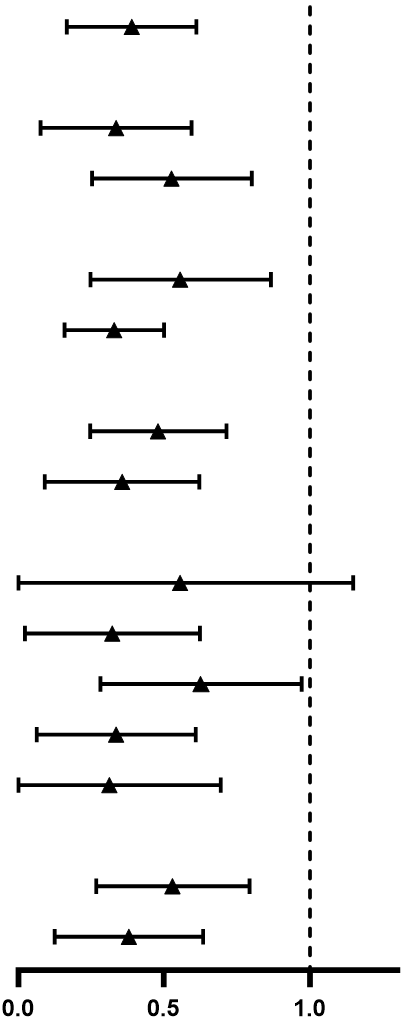**CVD mortality** | P value |
| --- | --- | --- | --- | --- | --- | --- |
| All patients | 0.75(0.45-1.23) |  | 0.252 | 0.35(0.19-0.63) |  | <0.001 |
| Age, y |  |  |  |  |  |  |
| <65 | 0.95 (0.43–2.05) |  | 0.900 | 0.28 (0.11–0.62) |  | 0.003 |
| ≥65 | 0.64 (0.34-1.20) |  | 0.167 | 0.48 (0.28-0.82) |  | <0.001 |
| Sex |  |  |  |  |  |  |
| Male | 0.51 (0.26–1.03) |  | 0.052 | 0.50 (0.28–0.89) |  | 0.018 |
| Female | 0.98 (0.47-2.25) |  | 0.941 | 0.31 (0.17-0.57) |  | <0.001 |
| BMI, kg/m2 |  |  |  |  |  |  |
| <30 | 0.61 (0.35-1.05) |  | 0.072 | 0.44 (0.27-0.73) |  | 0.001 |
| ≥30 | 0.93 (0.46-2.09) |  | 0.926 | 0.29 (0.13-0.65) |  | 0.004 |
| Race and ethnicity |  |  |  |  |  |  |
| Mexican American | 0.34 (0.10-1.44) |  | 0.312 | 0.32 (0.12-1.23) |  | 0.200 |
| Other Hispanic | 0.23 (0.09–1.08) |  | 0.132 | 0.23 (0.08-0.66) |  | 0.014 |
| Non-Hispanic White | 0.76 (0.38-1.51) |  | 0.433 | 0.56 (0.32-1.00) |  | 0.050 |
| Non-Hispanic Black | 0.75 (0.29–1.92) |  | 0.550 | 0.26 (0.11–0.64) |  | 0.003 |
| Other Race | 0.83 (0.33–2.65) |  | 0.845 | 0.13 (0.06–0.75) |  | 0.023 |
| Diabetes status |  |  |  |  |  |  |
| Diabetes | 0.70 (0.39-1.27) |  | 0.239 | 0.49 (0.29-0.81) |  | 0.006 |
| Pre-diabetes | 0.82 (0.34-1.95) |  | 0.654 | 0.32 (0.16-0.66) |  | 0.002 |
|  |  |  |  |  |  |  |

Adjusted hazard ratios for cancer mortality and CVD mortality in participants with DI-GM ≥6 and no sleep disorders, compared to participants with DI-GM 0-3 and sleep disorders, stratified by age, sex, BMI, race and ethnicity and diabetes status.
